# Initial findings of *striatum* tripartite model in OCD brain samples based on transcriptome analysis

**DOI:** 10.1038/s41598-019-38965-1

**Published:** 2019-02-28

**Authors:** Bianca C. G. Lisboa, Katia C. Oliveira, Ana Carolina Tahira, André Rocha Barbosa, Arthur Sant’Anna Feltrin, Gisele Gouveia, Luzia Lima, Ana Cecília Feio dos Santos, David Correa Martins, Renato David Puga, Ariane Cristine Moretto, Carlos Alberto De Bragança Pereira, Beny Lafer, Renata Elaine Paraizo Leite, Renata Eloah De Lucena Ferretti-Rebustini, Jose Marcelo Farfel, Lea Tenenholz Grinberg, Wilson Jacob-Filho, Euripedes Constantino Miguel, Marcelo Queiroz Hoexter, Helena Brentani

**Affiliations:** 1 Faculdade de Medicina FMUSP, Universidade de Sao Paulo, Sao Paulo, SP Brazil; 2 Inter-institutional Grad Program on Bioinformatics, University of Sao Paulo, Sao Paulo, SP Brazil; 3 Center of Mathematics, Computation and Cognition, Federal University of ABC, Santo Andre, SP Brazil; 4 Academic Research Organization–Hospital Israelita Albert Einstein, Sao Paulo, SP Brazil; 5 Memory and Aging Center University of California, San Francisco, USA

## Abstract

Obsessive-compulsive disorder (OCD) is a psychiatric disorder characterized by obsessions and/or compulsions. Different striatal subregions belonging to the cortico-striato-thalamic circuitry (CSTC) play an important role in the pathophysiology of OCD. The transcriptomes of 3 separate striatal areas (putamen (PT), caudate nucleus (CN) and *accumbens nucleus* (NAC)) from *postmortem* brain tissue were compared between 6 OCD and 8 control cases. In addition to network connectivity deregulation, different biological processes are specific to each striatum region according to the tripartite model of the striatum and contribute in various ways to OCD pathophysiology. Specifically, regulation of neurotransmitter levels and presynaptic processes involved in chemical synaptic transmission were shared between NAC and PT. The Gene Ontology terms cellular response to chemical stimulus, response to external stimulus, response to organic substance, regulation of synaptic plasticity, and modulation of synaptic transmission were shared between CN and PT. Most genes harboring common and/or rare variants previously associated with OCD that were differentially expressed or part of a least preserved coexpression module in our study also suggest striatum subregion specificity. At the transcriptional level, our study supports differences in the 3 circuit CSTC model associated with OCD.

## Introduction

Obsessive-compulsive disorder (OCD) is a psychiatric disorder characterized by obsessions and/or compulsions that are time consuming, distressing, or impair daily function and are not the direct result of a medical condition or substance use; the worldwide prevalence of OCD is 2–3%^1^. Family studies revealed OCD aggregation patterns; according to twin studies, the heritability of OCD is approximately 40%^2,3^. More recently, genome-wide association studies (GWAS)^4,5^ suggested that common variation in the heritability of OCD is between 25 and 30%, indicating an important contribution of single nucleotide polymorphisms (SNPs) with a minor frequency allele (MAF) of 5%. Meta-analysis of GWAS in OCD cases and controls^6^ as well as GWAS in obsessive-compulsive symptoms (OCS) in a population cohort^7^ studying polygenic risk scores (PRS) corroborate the importance of common variants explaining the phenotypic variance in OCD. Not all GWAS have found SNPs that are significant at the genomic level, but all have found marginally associated SNPs. Some of these SNPs are characterized as methylation quantitative trait loci (mQTLs) and expression quantitative trait loci (eQTLs) in brain areas^4,5^, while ENCODE/ROADMAP data suggested that other SNPs located in genome regions have regulatory potential^6^. Copy number variations (CNVs) as well as exome studies examining a higher burden of *de novo* variations for the involvement of very rare variations in OCD have also been performed^8–11^. Using an innovative statistical approach and integrating information from animal studies and targeting both coding and regulatory regions, Hyun Ji Noh *et al*.^12^ recently found functional variants associated with OCD. Based on Gene Ontology (GO) enrichment analysis and gene network analysis performed in the majority of the above studies, glutamate signaling, synaptic connectivity, and cortico-striato-thalamic circuitry (CSTC) are important in OCD pathophysiology.

Three smaller circuitries from different striatal subregions encompassing CSTC may play an important role in the pathophysiology of OCD^13,14^; the main characteristics of CSTC are the innervation of the frontal cortex towards the *striatum* (caudate *nucleus* (CN), putamen (PT) and *accumbens nucleus* (NAC))^14,15^. Each small circuitry has specific characteristics, including affective and limbic, cognitive and dorsal associative, and ventral and motor. Additionally, the relation between the brain regions, paradigms and symptoms of OCD have been explored by neuropsychological tests associated with neuroimaging investigations^16^. Accordingly, distinct emotional or cognitive impairments associated with OCD have been described with its brain signatures^17^.

In addition to symptomatic evidence involving different *striatum* areas associated with OCD, the *striatum* tripartite model and connectivity were defined by gene expression^18^. This validation of these small circuits has been demonstrated by delineating distinct *striatum* subregions based on connectivity using diffusion-weighted imaging (DWI) data. The authors parcellated *striatum* masks by grouping seed voxels with similar profiles of extrinsic whole-brain connectivity using k-means clustering. Then, the authors showed that these *striatum* subregions can be distinguished with high accuracy based on their gene expression profile. Dopamine receptor signaling and response to amphetamine were important sources of transcript variation separating the dorsal and ventral subregions of the *striatum*, while transcripts associated with glutamate secretion and metabolic processes separated the caudal subregion^18^. A recent paper compared the transcriptome of brain *striatum* subregions from controls and cases of Tourette syndrome (TS), which is often comorbid with OCD and has also been associated with CSTC^19^. In differentially expressed genes (DEGs) and coexpression module analyses, the authors found enrichment for interneuron signaling, neuronal catabolism, microglia signaling and astrocyte metabolism, but they analyzed the CN and PT together^19^.

As different areas of the *striatum* have transcriptome signatures and each area is more associated with a different portion of CSTC involved in the pathophysiology of OCD, we expected to find specific molecular profiles deregulated in *striatum* subregions by comparing OCD cases and controls. At the transcriptional level, these findings could corroborate and better explain the participation of these subregions in different circuitries involved in OCD. OCD is a polygenic multifactorial disorder characterized by multiple affected genes working in gene networks; thus, using only single measures of DEGs cannot reveal deregulation of the activity observed in complex systems^20^. Accordingly, we searched DEGs and nonpreserved coexpressed modules to explore quantitative and qualitative differences in the *striatum* tripartite model in OCD. In addition, we determined if genes previously associated with OCD by prior large-scale genomic studies were represented in different striatum subregion comparisons, contributing to possible functional roles of different genetic variants. To our knowledge, this report represents the first striatal postmortem OCD transcriptome study.

## Results

### General population characteristics

Samples were collected between October 2008 and June 2013. A total of 109 cases were screened as potential cases of psychiatric disorders. Seventy-two cases were assigned to the psychiatric group, and 37 cases were placed in the control group. Within the psychiatric group, the final diagnosis was OCD in 22 cases, and the remaining 50 cases were diagnosed with psychiatric disorders (bipolar disorder 19, major depression 16, TS 10, schizophrenia 2 and others 3) (Supplementary Fig. 1). Of the 22 OCD cases, 8 had the best estimate diagnosis and all *striatum* areas, but only 6 OCD cases had viable tissue for our investigation. Finally, we selected 8 controls (Table 1) for these 6 OCD cases matched by age, sex and laterality that had CN, PT and NAC subregion samples available (Supplementary Table 1). Supplementary Table 2 presents descriptions of all OCD cases.

**Table 1:** Demographic characteristics of obsessive-compulsive disorder (OCD) cases and controls (n = 14).

Variable	Parameters	OCD (ss = 6)	Control (ss = 8)	p-value
Age (years)*	Median	79.0	74.5	0.64
	Mean (SE)	79.8 (5.1)	74.1 (4.8)	
Sex n (%)**	Female	2 (33)	3 (38)	0.87
	Male	4 (67)	5 (62)	
Hemisphere n (%)**	Left	4 (67)	4 (50)	0.67
	Right	2 (33)	4 (50)	
Education in years*	Median	2.0	4.0	0.29
	Mean (SE)	2.7 (1.3)	5.5 (1.9)	
Alcohol abuse (%)**	Never	5 (83)	6 (75)	0.71
	Yes	1 (17)	2 (25)	
Tobacco abuse (%)**	Never	3 (50)	2 (25)	0.34
	Yes	3 (60)	6 (75)	
***Postmortem*** interval in hours*	Median	15:20	14:27	1.00
	Mean (SE)	15:26 (1:02)	14:51 (1:05)	
NPI n (%)**	Nil	2 (33)	7 (87)	0.05
	Positive	4 (67)	1 (13)	
Volume (ml) 1 missing in each arm*	Median	1100	1192	0.1
	Mean (SE)	1078 (35)	1283 (116)	
Mass (g) 1 missing in each arm*	Median	1162	1220	0.56
	Mean (SE)	1152 (56)	1182 (47)	

(%)* Test for median; (%)** test for log odds ratio; ss = sample size; n = frequency; NPI = Neuropsychiatric Inventory.

### Differential expression

The transcriptome of 42 brain samples of 3 *striatum* regions (CN, PT and NAC) from 6 OCD cases and 8 controls was investigated using high-throughput technology, resulting in 2.57 billion paired-end reads (avg. 61 million per sample). A total of 2.42 billion (92.5%) reads were aligned to the genome. Differential expression in each striatal region was obtained according to the expression of genes in each region, including CN (n = 44,815, with 17,972 Ensembl genes), NAC (n = 45,701, with 18,126 Ensembl genes) and PT (n = 45,470 with 17,886 Ensembl genes). Most parts of the assembled transcriptome were from unannotated regions comprising approximately 40% of our dataset. According to the Ensembl annotated dataset, 26% of the transcriptome was protein-coding genes, 3.5% lincRNAs and 3% antisense (Supplementary Fig. 2). Considering the small sample size in this study (6 OCD cases and 8 controls), we investigated the extent to which the results could be affected by the interindividual differences in gene expression between the donors. We used our dataset to calculate sample-specific weights using voom in the limma package in R^21^. According to Liu and collaborators^22^, samples with higher expression abundance are more robust measures; thus, they carry greater weight in expression analysis. Ideally, sample-specific weights are approximately one. In this study, we used the Surrogate Variable Analyses (SVA) package, which identifies sources of variation unaccounted for in a study. We used voom to calculate the weights with and without SVA variables (SVs) to determine whether SVs could mitigate the different sample weights in analyses. Sample-specific weight calculations were performed using a model including SVs (SVmodel) and a model that did not account for SV variables (NAmodel). For the CN dataset, compared with the NAmodel (mean = 1.28, var = 0.62, median = 1.23), the SVmodel resulted in sample weight variation (mean = 1.04, var = 0.078, median = 1.11). Therefore, sample-specific weights remained; however, including SV in the model resulted in a decrease in weight variation, leading to more homogeneous weights. No statistically significant difference was observed in weight distribution (ks = 0.1549). For the NAC dataset, both distributions of sample-specific weights were similar (Kolmogorov-Smirnov = 0.9205), although the SVmodel (mean = 1.03, var = 0.061, median = 1.09) resulted in slightly higher weights slightly than did the NAmodel (mean = 1.02, var = 0.041, median = 0.99). For the PT dataset, the SVmodel (mean = 1.03, var = 0.073, medina = 1.02) was slightly better than the NAmodel (mean = 1.05, var = 0.12, median = 0.99), which resulted in lower sample weight variation, with no differences between the two weight distributions (ks = 0.9205). Although the effect of sample-specific weight remained in the CN dataset, the use of SVs in the model could contribute to more homogeneous weights. In this way, the bias of sample weights was small and would not impact further analyses.

Differential expression analysis with adjusted p ≤ 0.05 resulted in 245 genes (186 ENSEMBL genes) in CN, 35 genes (20 ENSEMBL genes) in NAC and 38 genes (24 ENSEMBL genes) in PT (Supplementary Tables 3–5). No common genes were found in all three areas, but genes observed in more than one comparison (n = 6 with 6 ENSEMBL genes) were concordant in differential expression levels according to log2FC (OCD/CON). In the biological process enrichment analysis in CN, 19 categories mostly associated with immune response, synapse transmission and ion transport were identified. The PT enrichment analysis showed (97 categories) predominantly biological process associated with cell response and proliferation in addition to metabolic regulation (Supplementary Table 6). We could not achieve any enrichment for NAC. Our goal in this work was to compare *striatum* areas between cases and controls and to compare differences between *striatum* areas to achieve a biologically meaningful understanding of the comparison between areas. Thus, we explored DEGs using a p-value ≤ 0.01, without adjustment for multiple testing. We identified 1127 genes (757 ENSEMBL genes) in CN, 310 genes in NAC (201 ENSEMBL genes) and 306 genes (193 ENSEMBL genes) in PT (Supplementary Tables 3–5).

**Figure 1:**
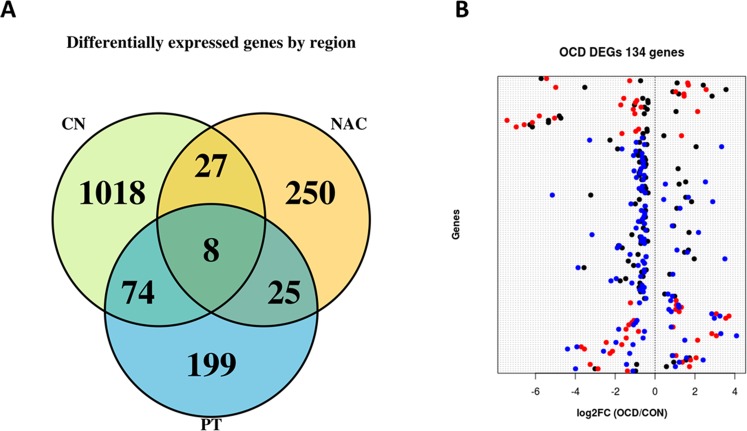
(**A**) Venn diagram of DEGs (p-value ≤ 0.01) of each striatal region CN, NAC and PT. (**B**) Dot blot of log2FC (OCD/CON) on the x-axis, and genes are represented on the y-axis. Each color corresponds to a striatal area CN (black), NAC (red) and PT (blue).

Genes observed in more than one comparison (n = 134 with 96 ENSEMBL genes, Fig. 1) were concordant in differential expression levels according to log2FC (OCD/CON) (Supplementary Table 7). Eight ENSEMBL genes, including three downregulated (NPAS4, RNUSA-1 and RP11.20J15.5) and five upregulated in OCD (TMPRSS5, bP-21264C1.3, bP-21264C1.1, and the two unannotated genes XLOC058328 and XLOC087511), were identified by comparing the analyses of three areas. Enrichment analysis of DEGs for functional categories (253 categories) showed predominant pathways related to immune response for CN but also synapse transmission and ion transport, as expected according to the more stringent criteria for DEGs in previous analyses. In PT, 68 categories were found. These categories corroborated the previous GO enrichment analyses, but cell response and regulation were more clearly associated with synapse function and neurotransmitter processes. For NAC, 14 categories were enriched. All these categories were associated with synapse and neurotransmission (Supplementary Table 6).

As we worked with bulk tissue, we also performed enrichment analysis for genes exclusive to different brain cell types^23^ (Table 2). To explore whether DEGs have been previously described in large-scale genomic OCD studies, we performed enrichment analysis using data from published GWAS^4,5^, exome^9^ and CNVs^8^. We also checked for DEGs in TS that could be comorbid with OCD^19^. We observed enrichment (p ≤ 0.05) only for TS genes in the three areas. Although none of the gene lists of common variations (all GWAS) or rare variations (exome and CNV studies) were enriched, some genes overlapped in specific OCD and control comparisons by region (Table 2 and Supplementary Table 8).

**Table 2:** Enrichment results for genes previously described in OCD studies and different brain cell types.

DEGs	Caudate Nucleus (CN)		Accumbens Nucleus (NAC)		Putamen (PT)	
	p-value	N matched genes	p-value	N matched genes	p-value	N matched genes
CNVs	0.1134	38	0.1930	11	0.8011	6
SNVs *de novo*	0.5010	1	1.0000	0	1.0000	0
GWAS	0.0514	10	0.7608	1	0.3971	2
Tourette syndrome	**0.0001**	159	**0.0001**	23	**0.0001**	35
Microglia	**0.0001**	108	0.5865	5	0.0290	10
Astrocytes	**0.0186**	32	0.3236	7	0.4389	6
Cortical Neuron 05	**0.0411**	12	**0.0001**	14	0.0988	4
Cortical Neuron 10	**0.0001**	51	**0.0001**	32	**0.0001**	20
Oligodendrocyte 01	0.1059	4	0.0867	2	0.0886	2
Oligodendrocyte 04	**0.0001**	14	1.0000	0	0.6662	1
Oligodendrocyte 2.5	0.0805	4	**0.0078**	3	0.0620	2

**Figure 2:**
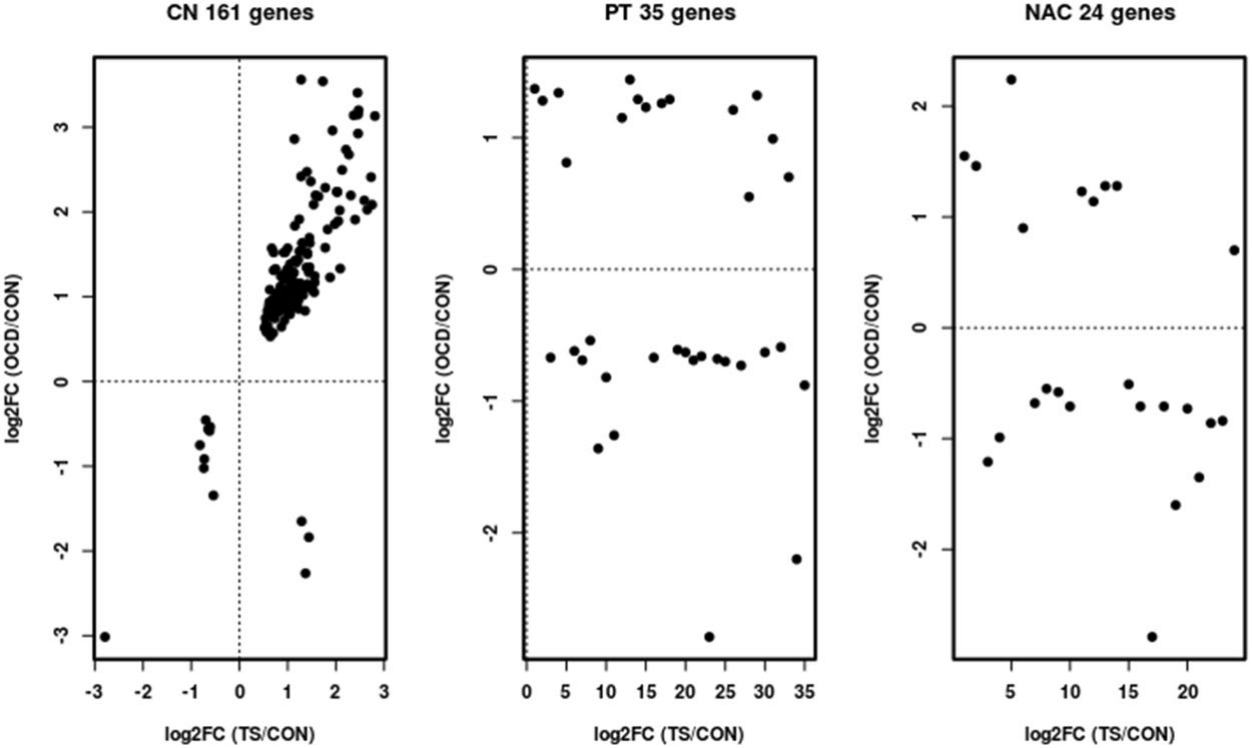
Scatter plots representing log2FC (TS/CON) of differentially expressed genes in Tourette syndrome in striatal regions (CN and PT) in the x-axis and log2FC (OCD/CON) of OCD DEGs in each striatal area CN, NAC and PT.

Finally, we compared our DEGs with those in a TS paper. Interesting DEGs (comparing CN and PT) in TS^19^ were enriched in all specific areas from this study, but differential expression levels were in the same direction in CN only; only three genes out of 161 were inverted (Fig. 2). For PT and NAC, only 15 (43%) and 9 (37.5%) genes had expression levels in the same direction, respectively.

### Coexpression analysis

**Figure 3:**
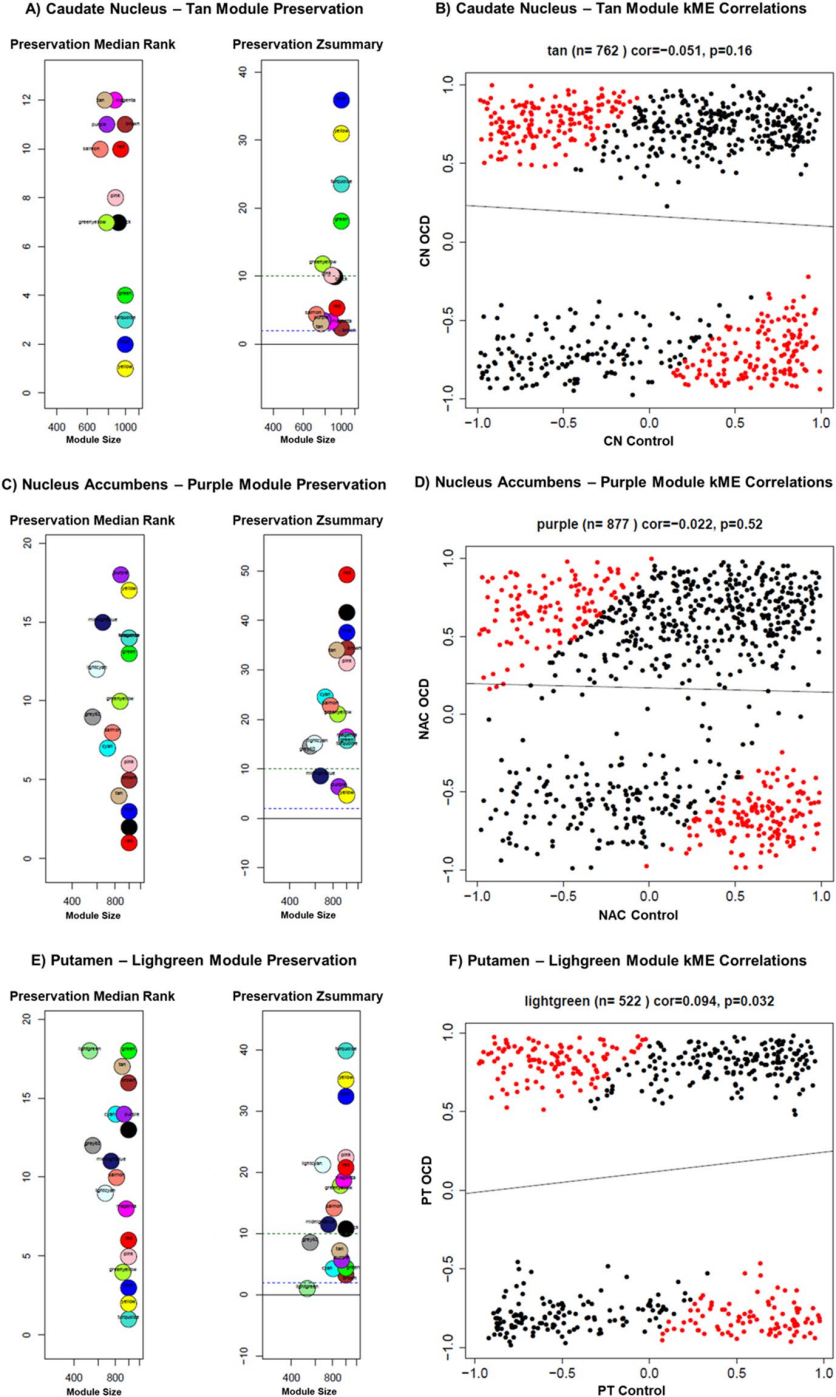
Preservation statistics–medianRank and Zsummary (**A**,**C**,**E**) and kME module correlation (**B**,**D**,**F**) of the least preserved module of the 3 brain regions, Tan (CN), Purple (NAC) and Lightgreen (PT). The preservation ranking for medianRank follows an ascending order (the least preserved module presents the highest scores), while for Zsummary, the least preserved modules have the lowest scores. For example, in (**E**), the Lightgreen module is the least preserved – its score in medianRank is 18 and 1 in Zsummary. For the correlation between the kME module, the control network is represented by the x-axis and OCD by the y-axis. The genes in red are the ones with the largest ratio of correlation between control and OCD groups; therefore, these genes are the most distinct by connectivity parameters.

To identify discrete modules according to gene expression, we performed coexpression module preservation analysis by using Weighted Correlation Network Analysis (WGCNA)^24^. To investigate which module genes were altered by comparing OCD and controls in each *striatum* area, we chose the least preserved module (the gene module that lost its network properties between the groups) according to medianRank, Zsummary and kME correlation. For CN data, we found 13 modules, and the Tan module (kME: n = 762; cor = 0.051, p = 0.16) was the least preserved. For NAC data, the Purple module (kME: n = 877, cor = 0.022, p = 0.52) was the least preserved from 16 modules; for PT, the Lightgreen module (kME: n = 522, cor = 0.094, p = 0.032) was the least preserved from 18 modules. Figure 3 shows the preservation module statistics, as well as the kME module correlation of the chosen least preserved module for each of the three brain regions (for medianRank and Zsummary scores, see Supplementary Table 9).

In all 3 analyses, all genes had a corresponding Ensembl gene annotation. Few gene ontologies were overrepresented in the least preserved modules in each striatal area (FDR ≤ 0.05). For NAC, positive regulation of the pathway-restricted SMAD protein phosphorylation biological process was enriched. In CN and PT, cellular component categories were enriched, specifically related to plasma membrane for CN (7 categories) and synapses for PT (10 categories) (Supplementary Table 10). In network analysis, some topological properties of nodes could characterize their relative importance in the network^25^. Hub genes are important genes because they are the most connected in the networks.

The complete list of hub genes comparing cases and controls of the least preserved modules of each *striatum* region are available in Supplementary Table 11. In PT, out of 522 genes in the module, we found 83 exclusive hubs in controls, 5 hubs in both cases and controls and 18 genes in exclusive hubs in OCD. In NAC, out of 877 genes in the module, we found 44 exclusive hubs in controls, 2 hubs in both cases and controls and 49 genes in exclusive hubs in OCD. In CN, out of 762 genes, we found 58, 6 and 48, respectively. To check whether DEGs were identified in the least preserved modules and associated with hub genes in each striatal area, we compared both results. In general, few genes were present in both analyses. In the CN module, there were 27 DEGs, including two hub genes in control (PIK3R5 and RP11-1319K7.1) and one (BAIAP2-AS1) in OCD. In the NAC module, there were only three DEGs, and none were hub genes. In PT, there were only 7 DEGs, and RPH3A was a hub gene in OCD only. Finally, we determined whether the least preserved modules of the 3 *striatum* regions have the same genes (Supplementary Figs 3 and 4). As expected, we did not observe any important overlap.

To check whether the less preserved modules from each area from the *striatum* have been described in large-scale genomic OCD studies, we performed the same analysis on DEGs with all datasets (Table 3). None were enriched, although overlaps were present in some specific areas, and astrocytes and cortical neurons were overrepresented in PT data only.

**Table 3:** Enrichment results for genes previously described in OCD studies and different brain cell types from WGCNA least preserved modules.

WGCNA	Caudate Nucleus (CN)		Accumbens Nucleus (NAC)		Putamen (PT)	
	p-value	N matched genes	p-value	N matched genes	p-value	N matched genes
CNVs	0.6897	24	0.9537	22	0.1908	22
SNVs *de novo*	1	0	0.4506	1	0.2982	1
GWAS	0.3103	6	0.7699	4	1	0
Tourette syndrome	0.7404	20	0.9642	18	0.5893	15
Adult Microglia	0.184	20	0.9883	10	0.9889	5
Astrocytes	0.1788	22	0.9716	13	**0.0057**	22
Cortical Neuron 05	0.9239	3	0.989	2	0.195	6
Cortical Neuron 10	0.9978	5	0.9889	8	**6.00E-04**	21
Oligodendrocyte 01	0.8266	1	0.3136	3	1	0
Oligodendrocyte 04	0.8996	2	0.9367	2	0.9268	1
Oligodendrocyte 2.5	0.8275	1	0.5891	2	0.6925	1

## Discussion

In this study, we used *postmortem* brain tissue to search for DEGs and nonpreserved coexpressed modules in OCD cases and controls. We showed that different DEGs as well as network connectivity deregulation were specific for each *striatum* region (CN, NAC and PT) by comparing OCD cases and controls. In addition, some genes associated with rare or common variation in published large-scale OCD genomic studies were differentially expressed in specific region comparisons.

The biological process enrichment analysis of DEGs showed that synaptic signaling was enriched for case-control comparisons in all areas, as well as the enrichment for neuron genes. Specifically, regulation of neurotransmitter levels and presynaptic processes involved in chemical synaptic transmission were shared between NAC and PT. Cellular response to chemical stimulus, response to external stimulus, response to organic substance, regulation of synaptic plasticity and modulation of synaptic transmission were shared between CN and PT. In addition, comparisons between areas showed that CN has a larger number of DEGs and that they have a greater overlap with DEGs from PT. Notably, common genes between different areas showed the same differentially expressed direction. Importantly, specific looking at the combination of DEGs from all areas showed that 58%, 15%, and 11% were exclusive to CN, NAC and PT, respectively. Additionally, only 0.5% of DEGs were common to all areas, suggesting that despite some shared biological processes, each area had exclusive transcriptomic signatures. The gene internexin neuronal intermediate filament protein alpha (INA), exclusive to NAC, has an important role in the transmission of information between neurons, in addition to being involved in neuronal morphogenesis for exoskeleton support^26,27^. The gene solute carrier family 5 (choline transporter) member 7 (SLC5A7), an exclusive DEG from NAC, was involved in cholinergic neuron functions^28^. In PT, we found some important genes, such as nitric oxide synthase 1 (NOS1) and synaptotagmin II (SYT2). The gene associated with modulator of synaptic activity, the (neuronal) NOS1 gene, is secreted by nerve terminals of brain regions that play a role in behavior and memory^29^. These findings related to synaptic alterations can be reinforced by the SYT2 gene, which is highly associated with synaptic vesicle membrane^30^. In CN, exclusive DEGs included gene regulator of G-protein signaling 7 binding protein (RGS7BP), nitric oxide synthase trafficking (NOSTRIN) and oligodendrocyte transcription factor 1 (OLIG1). RGS7BP, a G protein that is an important component of a chemistry system, can extend the excitability or inhibitory mechanism of synaptic transmission in postsynaptic neurons^31^. Similarly, the NOSTRIN gene can promote long-term neuronal transmission^29^. The OLIG1 gene plays a role in the maturation of the central nervous system and is directly associated with the development of oligodendrocytes^32^. Oligodendrocyte cells protect and support neurons, but new evidence in animal models shows that oligodendrocyte death can be associated with demyelination and an immediate immune response^33^. Thus, acute and chronic inflammation pathways are closely related to brain injuries and OLIG1 imbalance^34^.

The coexpression module analysis as well as the least preserved modules corroborate differences between areas. By comparing OCD cases and controls in each area, genes from the least preserved coexpressed module showed that 33.7%, 38.9%, and 23.4% of genes were exclusive to CN, NAC and PT, respectively, and none were common to all areas. Among the overlapping genes in the least preserved module in each area, CN shared more genes with NAC. Although PT did not present a greater number of DEGs, it had the most gene-gene correlations brooked between cases and controls in the coexpression module analysis, suggesting important gene network deregulation in OCD. Interestingly, in the cell enrichment analysis, astrocytes were enriched for PT DEGs. In animal models, astrocytes in striatal regions are associated with the dysfunction of calcium channels, contributing to synaptic deregulation of glutamatergic signaling^35^. We identified the D-amino acid oxidase (DAO) gene as a hub gene in CN. This gene plays an important role as a modulator of D-serine metabolism and consequently in the activity of the N-methyl-D-aspartate (NMDA) receptors^36^. NMDA is the most important receptor of the glutamatergic system. The SLC6A7 gene, which is also a hub gene to OCD in CN, is a gene from gamma-aminobutyric acid (GABA) neurotransmitter family^37^. The GO enrichment analysis based on the coexpression gene modules in NAC revealed positive regulation of SMAD protein phosphorylation by bone morphogenetic protein (BMP) genes, which is important to maintain the homeostasis of tissue, that plays essential roles in neurotransmitter specifications^38^. The BMP4 gene can induce a dopaminergic phenotype in mouse striatal neurons^39^.

The limited overlap of DEGs and the least preserved module genes in each striatal area confirmed the ability of the analyses^20^ to search for differences between OCD cases and controls in distinct *striatum* areas. Moreover, both analyses confirmed the specificity of the transcription signatures of the *striatum* areas previously described by Parkes^18^. Parkes also showed enrichment of the discrimination between dorsal and ventral subregions of the *striatum* for dopamine receptor signaling, whereas genes that can accurately classify the caudal subregion of the *striatum* were enriched for glutamate secretion. In our results, these differences were maintained in the comparison between OCD cases and controls.

The involvement of the immune system in OCD has long been suggested^40^. Kumar and colleagues^41^ found a rich association of microglial-activated inflammation and increased bilateral nucleo in TS. Recent robust outcomes demonstrated neuroinflammation in main areas from the CSTC in OCD cases^42^. Both studies support previous studies that found “pediatric autoimmune neuropsychiatric disorders associated with streptococcus” (PANDAS) by PET^43,44^. One interesting point revealed by our analyses is that biological processes specifically enriched for the immune system and cytokines appear in CN only. In CN, the categories related to cell adhesion molecules, which are strongly related to the immune system, were enriched. Dysregulation in these categories was also observed in methylation analyses comparing OCD and control^45^. Interestingly, CN showed microglial enrichment. OCD and TS frequently cooccur in individuals. There is evidence for shared OCD/TS genetic risk from family studies. GWAS joint analyses of TS and OCD suggested a higher burden of known pathogenic neurodevelopmental deletions in OCD/TS cases than in controls but not an overall higher burden of pathogenic CNVs. Alternatively, cross-disorder polygenic analyses based on PRS showed evidence for genetic heterogeneity between OCD and TS and suggested that OCD with and without chronic tics have different genetic architectures^46,47^. We compared our DEGs between cases and controls in each *striatum* area with a published study of DEGs from TS cases and controls performed with both PT and CN brain samples^19^. Significant enrichment of TS DEGs in all 3 areas of the *striatum* in our datasets was observed, but DEGs in TS were in the same direction of differential expression levels as OCD DEGs in CN. For PT and NAC, only 15 (43%) and 9 (37.5%) genes were in the same direction of expression levels. Therefore, deregulation of CN genes could be part of the specific genetic architecture shared by some TS and OCD cases and associated with microglial functions, including failures in neuroprotection, lack of support for neuronal survival, and abnormalities in synaptic pruning^48^.

Large-scale genomic OCD studies implied PRS as well as single very rare variations with larger effect sizes represented by SNVs and CNVs. In these studies, mQTLs, eQTLs and brain expression databases were compared, implying possible transcriptomic alterations and supporting an integrated model for OCD^49^. Accordingly, we searched different variations previously associated with OCD in our transcriptome analyses. Neither common nor very rare class variations were enriched in DEGs or least preserved coexpression modules in any striatal area between OCD cases and controls in this study. Jaffe *et al*.^50^ selected SNPs based on candidate gene studies for eating disorders (EA) and OCD from the literature. These authors showed the association of these SNPs with gene expression across the lifespan in the prefrontal cortex (PFC) among controls. When the authors used brain PFC samples from patients with EA and OCD/obsessive-compulsive personality disorder or tics and compared them with samples from controls, they found some DEGs, but none of the risk SNPs were eQTLs or associated with gene expression. Jaffe and colleagues^50^ argued that larger sample sizes would be necessary. Our small sample size probably explains our results, even though some genes harboring common and/or rare variants previously associated with OCD appeared in our gene sets. CNV studies uncovered 113 genes in at least one *striatum* comparison. Six (5.3%) out of 113 genes appeared in two *striatum* regions. From the SNV list, one gene was also represented in each *striatum* area, specifically WW domain containing E3 ubiquitin protein ligase 1 (WWP1) in NAC, CCDC108 in PT and complement C3b/C4b receptor 1 (CR1) in CN. Twenty-two genes were shared between GWAS and our gene sets. Only protein phosphatase 1 regulatory subunit 15A (PPP1R15A, 4.5%) was present in two different regions, CN and PT. Considering all genome studies and all gene sets from our study, 27% (37) were exclusive DEGs or in the least preserved coexpression gene modules in NAC, 17% (24) in PT, 51% (70) in CN and only 5% (7) in at least two *striatum* areas, confirming area specificity. The WWP1 gene is highly related to protein degradation and RNA transcription^51^ that is related to neurotransmitter imbalance. Thus, the PP1R15A gene, found in CN and PT, encodes proteins that contribute to stabilization of protein synthesis after stress exposition^52^. The specific gene in CN confirms that changes in the immune system can be related to OCD, and the CR1 gene encodes proteins with important functions in the activation of immune complexes, such as the neurabin 2 gene (PPP1R9B), found in CN and GWA studies, which is involved in the NK immunological synapse^53^. We also highlighted the synaptoporin gene (SYNPR), found in GWAS and a DEG in PT. SYNPR encodes a membrane protein involved in cholinergic imbalance and behavior, motor, cognition dysfunctions^54^, most likely affecting the nicotinic receptor in PT. The NSF attachment protein beta (NAPB) gene plays a role in the fusion of vesicles in presynaptic membranes^55^. Interestingly, the follistatin-like 5 (FSTL5) gene, previously implicated in cancer^56^ but expressed in the human brain^57^, was found in NAC and CNV studies. FSTL5 is expressed in cortical neurons and involved in Wnt/β-catenin signaling^58^. In addition, FSTL5 is important in cell development, synaptic transmission and plasticity^59^.

Importantly, the limitations of this study must be noted. The first limitation is the small sample size and relative heterogeneity of clinical symptoms. However, collecting OCD *postmortem* brains, maintaining high-quality frozen specimens and performing reliable clinical assessments are difficult. Notably, multiple steps with different trained personnel were performed to reach the psychiatric diagnosis. Although some Neuropsychiatric Inventory (NPI) items were abbreviated for some individuals, the assessment by more than one psychiatrist excludes any comorbidity. We also took care at all stages to ensure high-quality samples, short *postmortem* intervals, good pH for cerebrospinal fluid, good quality of the RNA integrity number (RIN) after RNA extraction and library preparation, using random samples distributed in the pooling library to obtain better results and reduce bias. Moreover, considering the small sample size in this study, we investigated the extent to which the results could be affected by interindividual differences in gene expression between the donors. Although a small effect of sample-specific weight existed in the CN dataset only, we used SVs in the model to prevent an impact on further analyses. Another important issue is that part of the assembled transcriptome was from unannotated regions comprising approximately 40% of our dataset and consequently was not used in the biological process enrichment analysis. As we used stringent quality control parameters and the same fact was observed in a TS transcriptome^19^ paper, we are likely capturing new *striatum* transcripts that could be very important to the OCD transcriptome. Finally, although we used age paired cases and controls, accessed different measures (anatomopathological and clinical information) associated with neurodegeneration and excluded any dubious samples, our study represents the transcriptome of individuals more than 65 years old, and our conclusions could be different for younger individuals.

In conclusion, this study is the first to explore the transcriptome of the separate *striatum* areas in OCD cases compared to that in controls. The results confirm the specificity of the transcription signatures of the *striatum* areas and better clarify possible different roles of each area in OCD pathophysiology at the transcriptional level. However, given the small sample size and heterogeneity of the OCD cases, this study is clearly the first step in efforts to sort the molecular basis of OCD in key brain regions. Future studies with more samples are necessary to understand this complexity.

## Methods

### Subjects and clinical evaluation

Brain samples were collected in the Sao Paulo Autopsy Service (SPAS) of the University of Sao Paulo and are part of the psychiatric collection of the Brain Bank of the Brazilian Aging Brain Study Group–University of Sao Paulo (BBBABSG). All subjects were 50 years of age or older and had no dementia, no factors related to hypoxia and no brain autolysis. The *postmortem* interval was less than 24 hours, and the minimum pH of cerebrospinal fluid was 6.0. The functional and psychiatric evaluations were performed by a family member or close caregiver who had at least weekly contact with the deceased and was available to answer the screening and semistructured questionnaires^60^. All family members gave written informed consent to participate in the study. The study itself, as well as the use of samples, was conducted with ethical approval granted by the FM-USP’s Institutional Review Board–Comissão de Ética para Análise de Projetos de Pesquisa (CAPPesq) under protocol number 0740/09, with all experiments performed in compliance with CAPPesq rules.

The clinical evaluation was performed in two steps. First, a screening to detect OCD and symptoms was performed according to DSM-IV. The screening consisted of the NPI, the short version of the structured clinical interview (SCID)^61^ and a short version of the Dimensional Yale-Brown Obsessive-Compulsive Scale (DY-BOCS)^62,63^. The Clinical Dementia Rating Scale (CDR)^64^ and semistructured questionnaires to detect Parkinsonism^65^, functionality and cognition were applied. During the second step, a psychiatric interview with an informant was performed, including the SCID for DSM-IV, the Yale-Brown Obsessive-Compulsive Scale (Y-BOCS)^62^ and the short version of the DY-BOCS. Thus, two psychiatrists who specialized in OCD performed two assessments for each case.

### RNA extraction and bioinformatics analysis

Dissected regions were stored at −80 °C until use. Total RNA was isolated from CN, PT, and NAC using a QIAsymphony RNA Kit (Qiagen, Germantown, MA, USA) according to the manufacturer’s instructions. The RIN was evaluated to check quality. The library was constructed with a TruSeq Stranded Total RNA Ribo-Zero Library Preparation Kit ((Illumina Inc., San Diego, CA, USA), and paired-end sequencing was performed with a HiSeq2500 (Illumina Inc., San Diego, CA, USA).

Quality control was performed using FASTQC (v.0.11.2)^66^ to check the sequence quality in 42 sequenced samples. Sequences were trimmed to eliminate poor base quality and adaptor contamination using FASTX (v.0.0.13)^67^. Sequences were mapped to Genome Reference Consortium Human build 38 (GRCh38) using Tophat2 (v.2.0.13)^68^ with a maximum of 2 mismatches. Cufflinks (v.2.2.1)^68^ was used to assemble transcripts using an Ensembl gene (GRCh38.78) as a reference annotation. Read counts were computed with the HT-seq^69^ algorithm with paired-end reads and gene attributes.

All statistical analyses were performed within the R environment (v.3.2.5)^21^. Transcripts assigned to more than one Ensembl gene, and any possible ribosomal RNA that bypassed depletion processes was removed from the data. To determine which genes were expressed, we converted read counts to counts per million (CPM) values (edgeR package - v.3.12.1)^70^, and transcripts with at least 0.3 CPM in 50% samples of a group (case or control) were used in downstream analysis. The data were normalized by the rlog function (DESeq2, v.1.10.1)^71^ to estimate hidden covariates using the SVA algorithm (v.3.18.0)^72^. The group was variables of interest, and sex, age and laboratory batch were adjustment variables. Differential expression analyses were performed within DESeq2, with the covariates sex, age, laboratory batch, and surrogate variables estimated by SVA included in the linear model.

Enrichment analyses of biological pathways from DEGs were performed in the web-based tool WebGestalt (http://www.webgestalt.org), using the Overrepresentation Enrichment Analysis (ORA) method and GO functional database. The reference lists used as background were all transcripts mapped as Ensembl genes. Functional categories with FDR ≤ 0.05 were considered enriched. Enrichment analysis for genes previously described in OCD studies and TS were performed using the Modular Single-set Enrichment Test tool^73^ within the R environment. The genes from each previous OCD study were acquired from the original papers and/or supplementary materials. From GWAS^4,5^, genes and eQTls for which the p-value was less than 10^*−*5^ were considered. The analyses of tissue specificity were based on genes from GWAS of animal model brains^23^. For these analyses, we performed orthologous conversion using an online tool DRSC ortholos (http://www.flyrnai.org/cgi-bin/DRSC orthologs.pl) with the following parameters: search field (Entrez ID, Gene name, Ensembl, HGNC and MGI ID). We selected all prediction tools (including Compara, eggNOG, HGNC, Homologene, Inparanoid, Isobase, OMA, OrthoDB, orthoMCL, Panther, and Phylome), and we selected the option, “return only best match when there is more than one match per input gene or protein”. From other OCD studies^9,19^, we based the list for our analysis on the best-scoring genes considered by the author.

### WGCNA parameters

WGCNA is an algorithm designed to evaluate connectivity differences between the coexpression network modules of two groups (i.e., case vs. control groups) based exclusively on expression data^24^. WGCNA uses Pearson’s correlation (rho) expression values between all gene pairs to calculate whether a pair of genes (nodes) are connected (edges) to each other. Nevertheless, the network must have a scale-free topology to conduct WGCNA analysis. The adjacency correlation matrix is elevated to a sequence of powers (N) with the intent to preserve only highly correlated values and to achieve a scale-free network topology. Choosing a minimum power (beta) value that implies a power-law degree distribution equivalent to a scale-free network (coefficient of determination *R*^2^ ≥ 0.8) is necessary.

In WGCNA, we used 23,713 transcripts with Ensembl gene annotations as input. Each brain region was analyzed separately. For each experiment, WGCNA removed transcripts for which more than 50% of the values were not available (NA) in both the case and OCD groups. In this way, CN represented a total of 20,960 genes, NAC 21,037 genes and PT 20,634 genes. For all three analyses (related to CN, NAC and PT regions), the chosen power (beta) value for both the control and OCD groups was 9. With the connectivity parameter set, all case and control datasets were analyzed separately. By using the connectivity parameters of the correlation matrix based on power (beta) = 9, the algorithm starts to cluster the pairs of genes based on Euclidean distance (d) between its correlation values, creating different modules (Topological Overlap Matrix–TOM). All TOM values range from 0 (closest) to 1 (farthest). We set a minimum size of 500 genes per module. Each module represents a different connected network of genes. Based on different connectivity parameters, WGCNA selects the first principal component of each module, identifying its value as a Module Eigengene (ME).

With the creation of all control and disease modules, we selected the control network as our reference network. In this way, the algorithm mapped all genes to each control network module and formed the same modules (with the same set of genes) in the OCD group. This analysis evaluates the difference between the connectivity of the same modules across different conditions (e.g., whether a set of hub genes in the control group remain hub genes in the OCD group). This analysis, Preservation Module Analysis^24^, is composed of two processes, medianRank and Zsummary statistics.

Zsummary uses a set of permutations (default value: 100 permutations) to analyze whether the connectivity parameters in the created modules are in fact significantly different from with modules (with the same size) created with random genes from the dataset. Zsummary provides a scale indicating the preservation level of a module; a Zsummary of 2 indicates a module with very little preservation, whereas 10 represents a well-preserved module. The medianRank does not use permutations; instead, it ranks modules according to connectivity parameters (such as separability, density and connectivity) based on the median value of each result. The results are presented in reverse order by Zsummary.

It is possible for medianRank and Zsummary to present distinct modules as the lowest preserved modules (partially because of the different methodologies of both statistics). In such cases, we used the correlation between the results of kME and the genes of the 3 lowest preserved modules of each analysis–the module that presents the most neutral correlation (closest to 0) is chosen as the least preserved module. We assume that the module with the lowest preservation represents the cluster of genes that are more altered between the OCD and control groups (considering the connectivity parameters). Thus, this set of genes would be the most promising to evaluate.

In addition to the preservation statistics, we analyzed the conservation of the hub genes of the least preserved modules. A gene with an absolute Pearson’s correlation ≥0.9 between its expression values and the kME value of each module was considered a hub.

## Supplementary information

Supplementary

Table S3

Table S4

Table S5

Table S6

Table S7

Table S8

Table S11

## Data Availability

The datasets generated during the current study are available in the SRA repository under Accession Number SRP127180, https://www.ncbi.nlm.nih.gov/bioproject/?term=PRJNA421175. All data generated in this study are included in the Supplementary Information.
