# Supplementary material for: Initial findings of striatum tripartite model in OCD brain samples based on transcriptome analysis
Source: Sci Rep. 2019 Feb 28;9:3086. doi: 10.1038/s41598-019-38965-1 (PMC6395771; doi:10.1038/s41598-019-38965-1)
Supplement: Supplementary file 1 — Supplementary [file 41598_2019_38965_MOESM1_ESM.pdf]

Initial findings of *striatum* tripartite model in OCD brain samples based on transcriptome analysis.

Bianca C. G. Lisboa[1,+], Katia C Oliveira[1,+], Ana Carolina Tahira[1], Andre Barbosa[1,2], Arthur Sant'Anna Feltrin[3], Gisele Gouveia[1], Luzia Lima[1], Ana Cecília Feio dos Santos[1], David Correa Martins-Jr[3], Renato Puga[4], Ariane Cristine Moretto[1], Carlos Alberto de Bragança Pereira[1], Beny Lafer[1], Renata Elaine Paraizo Leite[1], Renata Eloah de Lucena Ferretti-Rebustini[1], Jose Marcelo Farfel[1], Lea Tenenholz Grinberg[1,5], Wilson Jacob-Filho[1], Euripedes Constantino Miguel[1], Marcelo Queiroz Hoexter[1], and Helena Brentani[1*]

[1]Faculdade de Medicina FMUSP, Universidade de Sao Paulo, Sao Paulo, SP, BR
[2]Inter-institutional Grad Program on Bioinformatics, University of Sao Paulo, Sao Paulo, SP, Brazil
[3]Center for Mathematics, Computation and Cognition, Universidade Federal do ABC, Santo Andre, SP, Brasil
[4]Academic Research Organization - Hospital Israelita Albert Einstein, Sao Paulo, SP, Brazil
[5]Memory and Aging Center University of California, San Francisco, USA
[*]helena.brentani@gmail.com
[+]These authors contributed equally to this work

Supplementary

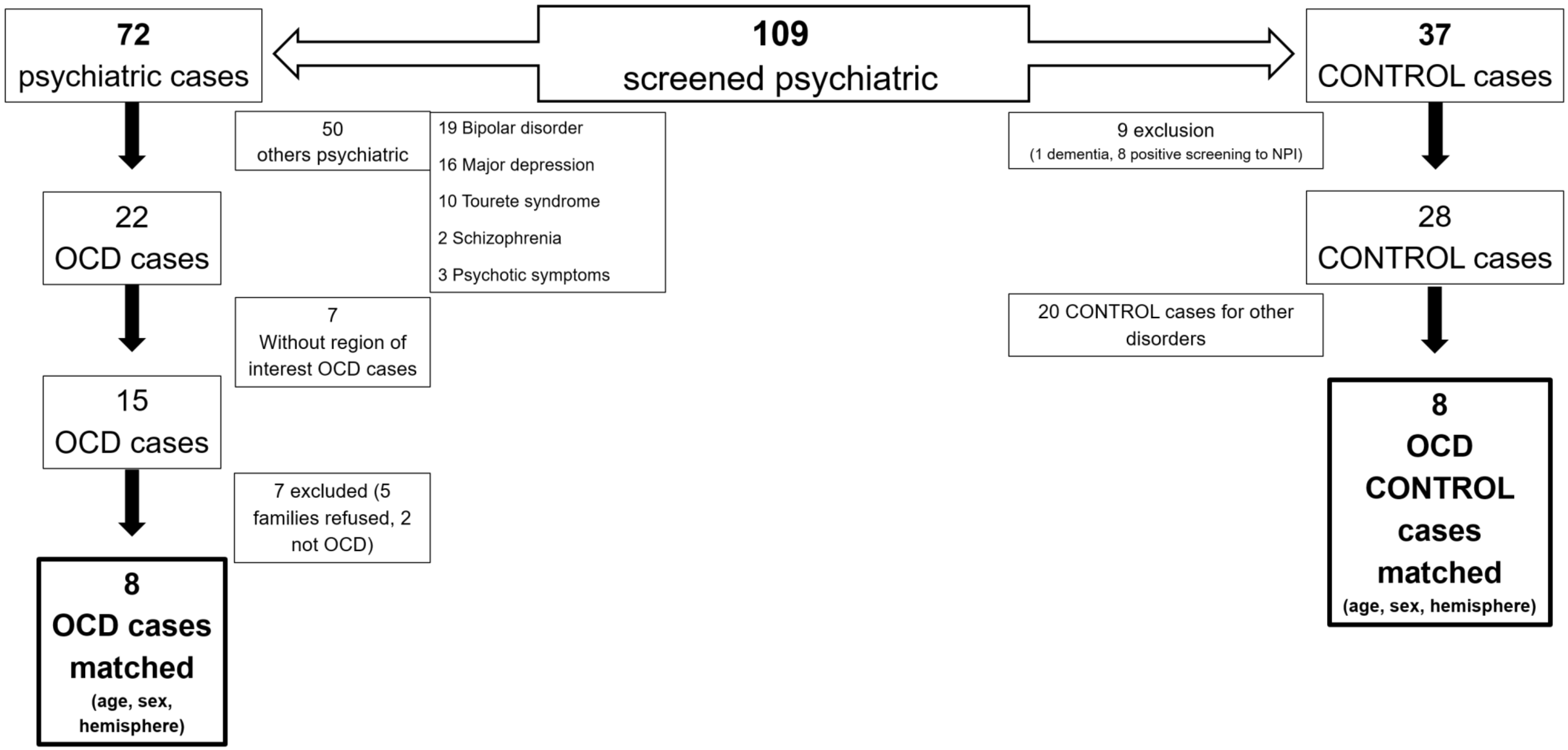

Supplementary Figure 1. Flowchart of samples collected and selected for this study.

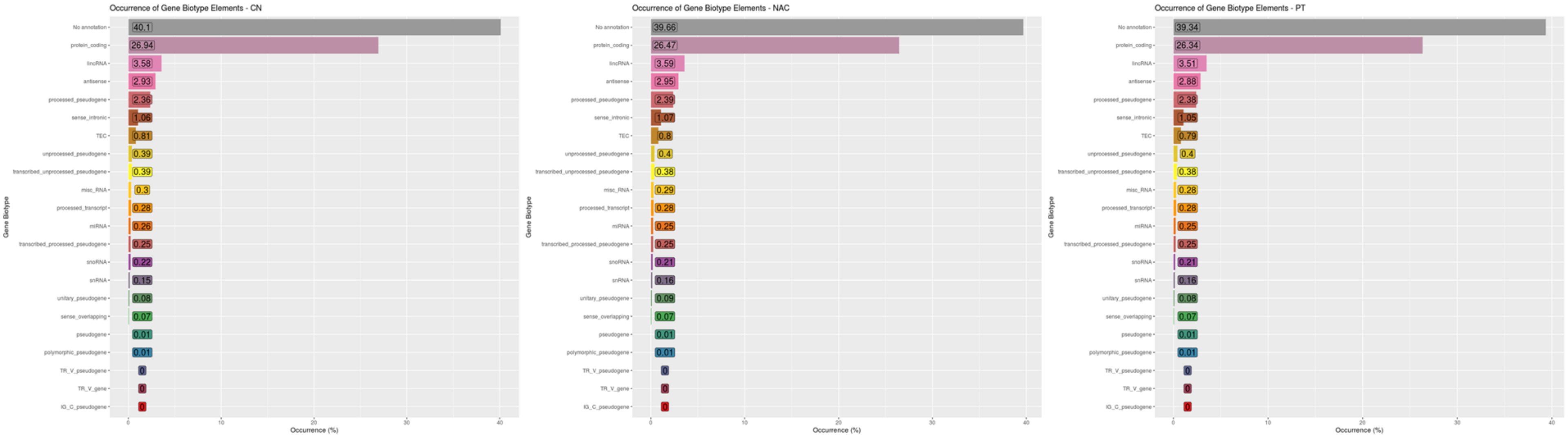

Supplementary Figure 2. Gene biotype composition of datasets from CN, NAC and PT assembled transcriptomes.

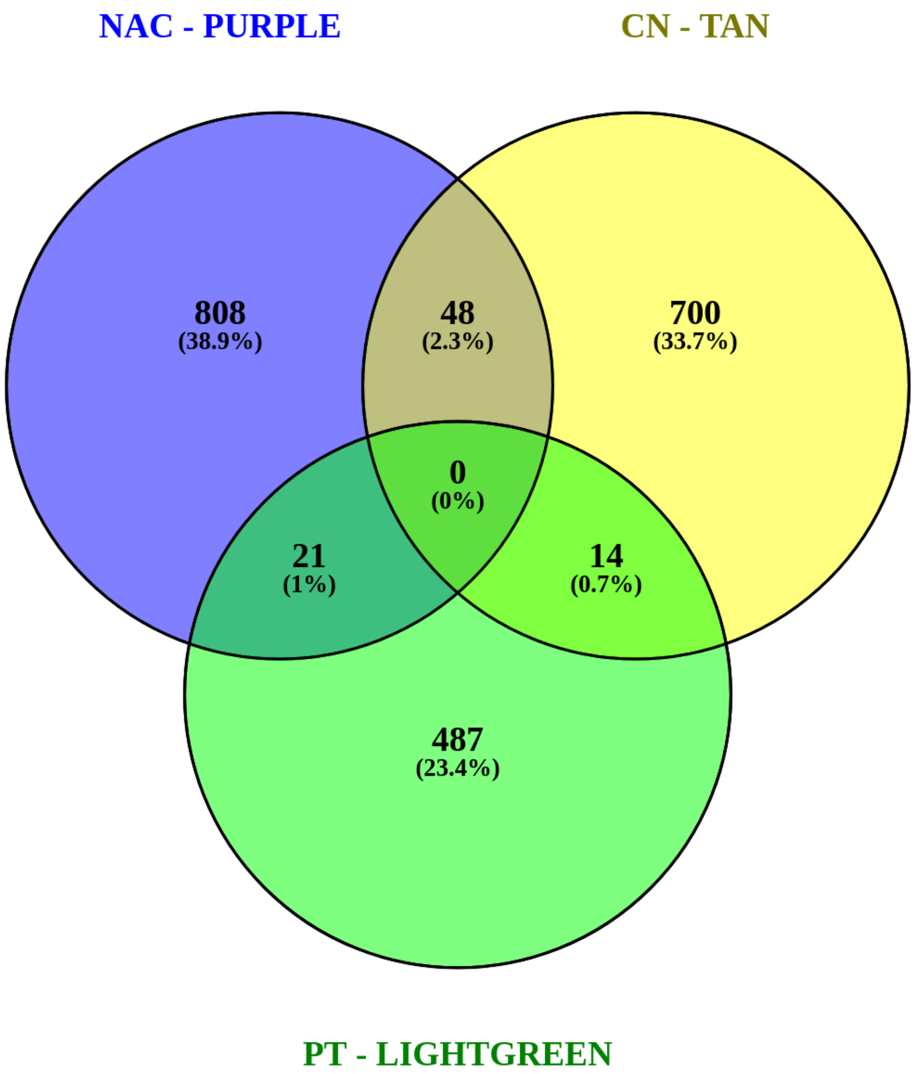

Supplementary Figure 3. Venn diagrams containing the intersection between all genes of the less preserved module of each region (CN, NAC and PT) according to preservation statistics and kME correlations.

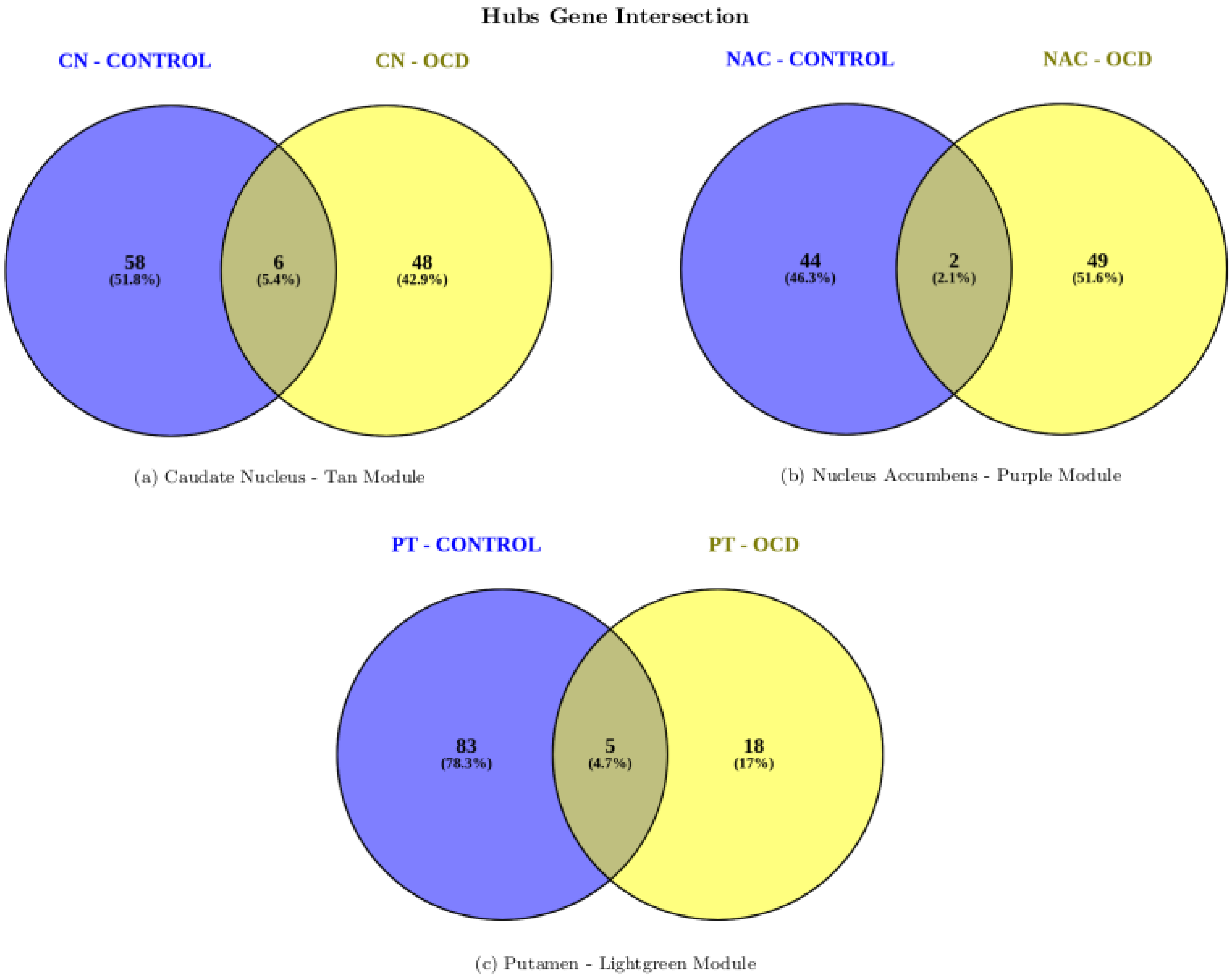

(a) Caudate Nucleus - Tan Module

(b) Nucleus Accumbens - Purple Module

(c) Putamen - Lightgreen Module

Supplementary Figure 4. Venn diagrams containing the intersection between the hubs in control and OCD networks between each region (CN, NAC and PT).

Table S1: Demographic characteristics of the study population (n=14)

ID	Sex	Age	Hemisphere	pH	IPM (hour)	Schoolar (years)	Sort of OCD symptoms	Cause of Death	Co-morbidity	Alkohol	Tabacco	Psychiatric Medication	CDR	IQCODE	NPI	NPI behavioral disturbances	SCIDD	SCIDM	Mpc (cm)	Mgo (cm)	Volume (mL)	Wheight	RIN_ CN	RIN_ NAC	RIN_ PT
CON-01	M	61	Right	6.5	12:35	15	NA	Heart attack	Systemic arterial hypertension, Cardiomegaly	Never	Yes	Not	0	3	0	#	Not	Not	57.0	35.4	1276	1335	8.3	8.0	8.0
CON-02	M	77	Left	6.8	17:20	11	NA	Acute peritonitis/cecum adenocarcinoma perforated	None	Never	Never	Not	0	3	0	#	Not	Not	#	#	1494	1186	10	8.9	9.6
CON-3	F	62	Left	6.6	12:45	0	NA	Pulmonary edema	Systemic arterial hypertension, Diabetes mellitus, Previous myocardial infarction	Never	Yes	Not	0	3	0	#	Not	Not	53.5	32.5	1192	1252	8.4	8.5	5.1
CON-04	M	82	Right	6.33	16:10	8	NA	Intra-abdominal haematoma/ruptured aortic aneurysm	Systemic arterial hypertension	Stopped	Stopped	Not	0	3	0	#	Not	Not	53.0	41.0	1100	1136	10	8.2	10
CON-05	F	98	Right	6.5	16:31	0	NA	Pulmonary edema	Systemic arterial hypertension, Diabetes mellitus	Never	Stopped	Not	0	3	9	appetite and eating abnormalities	Not	Not	49.5	33.5	850	914	10	8.8	9.9
CON-06	M	83	Left	7.0	12:34	4	NA	Pulmonary edema	Systemic arterial hypertension, Generalized atherosclerosis, Ischemic cardiomyopathy	Yes	Stopped	Not	0	3	0	#	Not	Not	58.2	35.0	#	#	9	7.4	8.3
CON-07	M	72	Right	7.0	19:54	2	NA	Acute myocardial infarction	Systemic arterial hypertension, Prostate cancer	Never	Never	Not	0	3	0	#	Not	Not	#	#	1882	1220	9.1	9.1	9.1
CON-08	F	58	Left	7.0	11:00	4	NA	Pulmonary thromboembolism	Ovarian cancer	Never	Yes	Not	0	3	0	#	Not	Not	53.0	53.0	1190	1232	7.7	9.1	6.3
OCD-02	M	77	Left	6.51	17:00	0	contamination/cleaning; compulsions to count	Advanced pancreatic carcinoma	Coronary atherosclerosis, Motor tics possible	Never	Never	Not	0	3	14	hallucinations[1]; aberrant motor behavior; night-time behavior disturbances	Not	Not	58.0	34.0	1100	1258	7.3	8.3	9.8
OCD-04	M	81	Right	7.0	13:55	1	ordering/arranging; aggression/disaster; compulsions of hoarding	Bronchopneumonia	Systemic arterial hypertension, Coronary artery disease, Congestive heart failure	Never	Never	Not	0	3	0	#	Presen t	Not	45.5	31.0	1100	1162	9.4	8.7	9.4
OCD-05	F	99	Left	6.43	15:51	0	compulsions of checking; contamination/cleaning; ordering/arranging; hoarding	Cardiac tamponade, Massive hemopericardium	Coronary atherosclerosis	Never	Stopped	Not	0	3	18	delusions[2]; agitation; aberrant motor behavior; night-time behavior disturbances; appetite and eating abnormalities	Not	Not	52.0	38.0	1000	956	9.4	5.8	8.7
OCD-06	M	86	Left	6.6	14:50	3	contamination/cleaning; obsessions about disasters/compulsions of checking	Pulmonary thromboembolism	Systemic arterial hypertension, Chronic obstructive pulmonary disease, Congestive heart failure, Prostate cancer	Never	Yes	Not	0	3	18	night-time behavior disturbances; appetite and eating abnormalities	Not	Not	53.0	36.0	988	1086	9.9	6.5	9.9
OCD-07	M	74	Right	8.0	19:11	8	obsessions about symmetry/'just-right' perceptions/compulsions to order/arrange; hoarding	Pulmonary edema	Systemic arterial hypertension, Hypertensive cardiopathy	Stopped	Yes	Not	0	3	49	agitation, dysphoria, anxiety, apathy, night-time behavior disturbances, and appetite and eating abnormalities	Not	Not	#	34.0	1200	1298	9.7	9.6	9.7
OCD-08	F	62	Left	7.2	11:50	4	obsessions of disasters; obsessions about symmetry/'just-right' perceptions/compulsions to order/arrange; miscellaneous obsessions/compulsions that relate to superstitions; hoarding	Pulmonary thromboembolism	Peripheral vascular insufficiency	Never	Never	Not	0	3	0	#	Not	Not	#	#	#	#	9.1	9.8	9.3

M: Male; F: Female; IPM: interval *postmortem*; CDR: Clinical Dementia Rating; IQCODE: Informant Questionnaire on Cognitive Decline in the Elderly; NPI: Neuropsychiatry Inventory; SCIDD: Structured clinic interview for depression; SCIDM: Structured clinical interview for mania; RIN_CN: RNA integration number from caudate nucleus tissue; RIN_NAC: RNA integration number from accumbens nucleus tissue; RIN_PT: RNA integration number from putamen tissue; #: no data

[1] Information very nonspecific. Family reported that the subject used to talk to himself (mild frequency and severity). Subject was never diagnosed with a psychotic disorder in life.

[2] Information very nonspecific from relatives. Family reported that the subject used to say that people wanted to steal him. Subject was never diagnosed with a psychotic disorder in life.

Table S2. Description of obsessive-compulsive symptoms of the OCD patients

Sample ID	OCD Symptoms
OCD-02	He washed objects and hands excessively (10-12 times per day, each washing used to take at least 10 minutes). He took long baths and worried about dirt (contamination/cleaning). Every morning, he had to count the seats before sitting down (compulsions of counting). He also had motor tics.
OCD-04	He locked all closets with padlocks to avoid other people from moving or arranging his things in a different way. He also had to check and recheck whether the padlocks were locked many times a day (ordering/arranging). He wore the same clothes many times to prevent “bad things” from happening (obsessions about disasters). He kept expired medications and useless broken objects and became anxious if he had to discard them (hoarding).
OCD-05	He used to open and close doors many times to verify that they were locked (compulsions of checking). He used to wash his hands excessively to keep them clean (contamination/cleaning). He was perfectionist and had to organize objects in his bedroom and in the kitchen in a specific way. If other people modified the way the objects were organized, he became very anxious (ordering/arranging). He also used to accumulate stuff (hoarding).
OCD-06	He was afraid of germs and being dirty. To avoid getting a disease, he used to wash his hands and face using a clean towel several times a day (contamination/cleaning). He had also several rituals to decontaminate the food he cooked. He had rituals of checking whether the gas was on or off and whether the windows were closed or opened to avoid something terrible (obsessions about disasters/compulsions of checking). A physician prescribed an antidepressant in the past, but he did not take it.
OCD-07	He had to arrange objects in the “right” way and it had to be “perfect”; otherwise, he became very anxious (obsessions about symmetry/‘just-right’ perceptions/compulsions to order/arrange). He also had a collection of useless objects on a shelf in the service area. He did not allow anyone to throw it away (hoarding).
OCD-08	She had to go out using the same door she entered to avoid something bad (obsessions about disasters). Objects had to be arranged in the “right” and “perfect” way (obsessions about symmetry/‘just-right’ perceptions/compulsions to order/arrange). She had superstitious magical beliefs in regard to number 3 (three tablespoons of rice, three sips of water, three pieces of meat) (miscellaneous obsessions/compulsions that relate to superstitions). She used to collect things without a clear purpose and became very anxious if someone would sort or throw the objects away (hoarding).

Table S9: WGCNA results (size of least preserved modules)

Region	Module	Module size (n)	medianRank	Zsummary	kME.cor	kME.p
CN	Tan	762	12	3	-0.051	0.16
NAC	Purple	877	18	6.4	-0.002	0.52
PT	Light green	522	18	1	0.094	0.032

Table S10: Enrichment functional categories of least preserved modules in each striatal area

GO	description	C	O	E	R	PValue	FDR	overlapGene
**CN – GO Celular component**								
GO:0044459	plasma membrane part	1871	59	36.44190556	1.61901523	7.7532E-05	0.01448481	362;399;799;914;1132;1524;1829;2044;2151;2315;2359;3001;3036;3037;3077;3645;3683;3693;3728;3743;3762;4224;4671;4864;5002;5754;6442;6534;6571;7070;7380;8323;8516;9019;9033;9365;10008;10077;10457;22801;22821;23166;23224;26266;27035;27133;29881;50937;51348;58494;60412;80758;84634;113655;117195;131890;154810;222962;374403
GO:0005887	integral component of plasma membrane	1141	41	22.22352445	1.84489189	8.0878E-05	0.01448481	362;799;914;1132;1524;2044;2151;2315;2359;3036;3037;3077;3645;3683;3693;3743;3762;4224;4864;5754;6534;6571;7070;8323;8516;9019;9365;10008;10077;10457;22801;23166;26266;27035;27133;50937;51348;58494;84634;113655;117195
GO:0008305	integrin complex	24	5	0.467453626	10.6962482	8.4304E-05	0.01448481	3683;3693;8516;10077;22801
GO:0031226	intrinsic component of plasma membrane	1185	42	23.08052277	1.81971615	8.9E-05	0.01448481	362;799;914;1132;1524;2044;2151;2315;2359;3036;3037;3077;3645;3683;3693;3743;3762;4224;4864;5754;6534;6571;7070;8323;8516;9019;9365;10008;10077;10457;22801;22821;23166;26266;27035;27133;50937;51348;58494;84634;113655;117195
GO:0098636	protein complex involved in cell	27	5	0.525885329	9.50777617	0.00015267	0.01987779	3683;3693;8516;10077;22801
GO:0030057	desmosome	20	4	0.389544688	10.2683983	0.0005315	0.04972917	1825;1829;3728;11187
GO:0009986	cell surface	521	22	10.14763912	2.16799196	0.00053472	0.04972917	914;1829;2044;3077;3683;3693;3762;4057;4063;5154;7070;7422;8321;8323;8516;9019;9033;10077;27133;84634;113026;282679
**NAC – GO Biological process**								
GO:0010862	positive regulation of pathway-restricted SMAD protein	34	7	0.766985946	9.12663399	8.7415E-06	0.04785088	94;652;656;657;2661;8200;9518
**PT – GO Cellular component**								
GO:0045202	synapse	596	28	11.70893761	2.39133566	1.6072E-05	0.01046316	323;1013;1128;1141;2263;2534;3688;4204;4735;5138;6833;6856;8497;9381;9479;9627;10235;11178;22895;29993;53335;57586;57657;116225;124925;126129;146395;221692
GO:0044463	cell projection part	740	31	14.53794266	2.13235124	5.0989E-05	0.01123273	323;1128;1259;1627;1770;3508;3685;3688;4735;6102;8120;8506;9479;10235;10566;10672;10979;11178;23265;25791;26160;27019;29993;55764;57657;81631;85452;114327;121441;124925;147700
GO:0042995	cell projection	1381	48	27.13094435	1.76919754	5.1764E-05	0.01123273	111;323;387;816;1128;1259;1627;1770;2534;3508;3685;3688;4289;4534;4735;4987;6102;8120;8506;9362;9479;10235;10566;10672;10979;11170;11178;22895;23108;23265;23603;25791;26160;27019;29993;55582;55764;57657;81631;85452;114327;117144;121441;124925;126129;147700;167691;255101
GO:0097458	neuron part	983	37	19.3118887	1.91591825	8.857E-05	0.01441477	323;816;1128;1259;1627;2534;3508;3688;4735;4987;5138;6602;6833;6856;8120;8193;8497;8506;9362;9381;9479;9627;10235;11170;11178;22895;23108;23265;25791;29993;55764;57586;57657;114327;124925;126129;147700
GO:0005874	microtubule	302	16	5.933052277	2.69675696	0.00030896	0.03420123	143;1770;1962;6760;10397;23332;27019;55177;55582;57509;64147;64837;81631;113220;147700;221035
GO:0005929	cilium	365	18	7.17074199	2.51020048	0.00031522	0.03420123	111;1259;1770;4735;6102;10566;26160;27019;55582;55764;81631;85452;114327;117144;121441;147700;167691;255101
GO:0005930	axoneme	72	7	1.41450253	4.94873629	0.00051373	0.03971673	1770;4735;26160;27019;81631;85452;114327
GO:0097014	ciliary plasm	72	7	1.41450253	4.94873629	0.00051373	0.03971673	1770;4735;26160;27019;81631;85452;114327
GO:0090544	BAF-type complex	20	4	0.392917369	10.1802575	0.00054908	0.03971673	6602;6760;8193;196528
GO:0015630	microtubule cytoskeleton	820	30	16.10961214	1.86224223	0.00070283	0.04575424	143;816;1770;1962;4735;6102;6760;7813;9700;9851;10397;23108;23265;23332;26160;27019;55177;55582;55764;57509;58490;64147;64837;81631;113220;114327;121441;147700;167691;221035

C: number of reference genes in the category; O: number of genes in the user gene list and in the category; E: expected number in the category; R: ratio of enrichment.
